# Right ventricular failure in septic shock: characterization, incidence and impact on fluid responsiveness

**DOI:** 10.1186/s13054-020-03345-z

**Published:** 2020-11-01

**Authors:** Antoine Vieillard-Baron, Amélie Prigent, Xavier Repessé, Marine Goudelin, Gwenaël Prat, Bruno Evrard, Cyril Charron, Philippe Vignon, Guillaume Geri

**Affiliations:** 1 Intensive Care Unit, Assistance Publique-Hôpitaux de Paris, University Hospital Ambroise Pare, Boulogne Billancourt, France; 2 Faculty of Medicine Simone Veil, Saint Quentin en Yvelines, France; 3 Inserm U1018, Center for Research in Epidemiology and Population Health (CESP), Faculty of Paris Saclay, Villejuif, France; 4 Intensive Care Unit, Brest University Hospital, Brest, France; 5 Intensive Care Unit, Limoges University Hospital, Limoges, France; 6 INSERM CIC 1435, Limoges University Hospital, Limoges, France; 7 Faculty of Medicine, University of Limoges, Limoges, France

**Keywords:** Right ventricular failure, TAPSE, Fluid responsiveness, Central venous pressure, Critical care echocardiography

## Abstract

**Objective:**

Incidence of right ventricular (RV) failure in septic shock patients is not well known, and tricuspid annular plane systolic excursion (TAPSE) could be of limited value. We report the incidence of RV failure in patients with septic shock, its potential impact on the response to fluids, as well as TAPSE values.

**Design:**

Ancillary study of the HEMOPRED prospective multicenter study includes patients under mechanical ventilation with circulatory failure.

**Setting:**

This is a multicenter intensive care unit study

**Patients:**

Two hundred and eighty-two patients with septic shock were analyzed. Patients were classified in three groups based on central venous pressure (CVP) and RV size (RV/LV end-diastolic area, EDA). In group 1, patients had no RV dilatation (RV/LVEDA < 0.6). In group 2, patients had RV dilatation (RV/LVEDA ≥ 0.6) with a CVP < 8 mmHg (no venous congestion). RV failure was defined in group 3 by RV dilatation and a CVP ≥ 8 mmHg. Pulse pressure variation (PPV) was systematically recorded.

**Interventions:**

None.

**Measurements and main results:**

In total, 41% of patients were in group 1, 17% in group 2 and 42% in group 3. A correlation between RV size and CVP was only observed in group 3. Higher RV size was associated with a lower response to passive leg raising for a given PPV. A large overlap of TAPSE values was observed between the 3 groups. 63.5% of patients with RV failure had a normal TAPSE.

**Conclusions:**

RV failure, defined by critical care echocardiography (RV dilatation) and a surrogate of venous congestion (CVP ≥ 8 mmHg), was frequently observed in septic shock patients and negatively associated with response to a fluid challenge despite significant PPV. TAPSE was unable to discriminate patients with or without RV failure.

## Introduction

Over the last decade, a few studies have highlighted the crucial role of right ventricular (RV) function in hemodynamic and respiratory diseases in critical care [1, 2]. However, RV failure is difficult to define in the critical care setting. A recent consensual definition proposed by different groups of experts characterizes RV failure as the association of a “significant” RV dilatation associated with systemic congestion [3–5]. This definition is mainly physiologically based, not strictly validated, and the experts did not propose any threshold for RV size or congestion using critical care echocardiography (CCE) and central venous pressure (CVP), respectively.

Fluid management in septic shock is crucial for prognosis, as fluid overload and high CVP are associated with a worse outcome [6]. It has been suggested that pulse pressure variations (PPV) could be observed despite altered fluid responsiveness due to RV failure [7, 8], though this is still questionable. Septic shock is expected to impair RV function through the development of septic cardiomyopathy, but the incidence of RV failure is unknown.

In a large series of patients with septic shock, our goals were then to report the distribution of RV size and CVP, to determine the incidence of RV failure and its impact on fluid responsiveness.

## Methods

### Patients

We did a post hoc analysis of the multicenter observational and prospective HemoPred study in which 540 ventilated patients admitted between 2012 and 2014 were included in five French ICUs (Amiens, Boulogne-Billancourt, Brest, Limoges, Tours and Suresnes) for circulatory failure. Transthoracic echocardiography (TTE) and transesophageal echocardiography (TEE) were systematically performed within the first 24 h of ICU admission [11]. The investigation conforms with the principles outlined in the *Declaration of Helsinki*. HemoPred was approved by the Ethics Committee of Limoges (no 85-2012-09). Among the 540 studied patients, 295 were admitted for septic shock, which was not characterized by the Sepsis-3 definition as the HemoPred study was designed before its publication. Diagnosis was based on a suspected infection responsible for sustained hypotension despite adequate fluid loading that required vasopressors, with associated clinical signs of tissue hypoperfusion (mottled skin, encephalopathy, oliguria for more than 2 h) that were confirmed by laboratory values (pH < 7.38 and base deficit > 5 mmol/L or lactate > 2 mmol/L or central venous oxygen saturation < 70%). One hundred and fifty-two patients met the lactate criteria as well as another hypoperfusion sign, while 115 did not meet the lactate criteria and 6 only met the lactate criteria.

### Clinical and laboratory data

The initial clinical data collected included socio-demographic data, biometric parameters, comorbidities, vital parameters (heart rate, systolic, diastolic and mean arterial blood pressure) and etiology of sepsis. The Sequential Organ Failure Assessment Score (SOFA) and Simplified Acute Physiology Score SAPS2 were recorded.

CVP was obtained by measuring the pressure at the end of a central catheter located at the level of the intrathoracic superior vena cava (SVC) at its entry into the right atrium. Bladder pressure was used as a surrogate of intra-abdominal pressure (IAP) (available in 260/282—92.2% patients), and PPV was calculated from the data obtained from the radial or femoral arterial catheter. Arterial blood gas analysis was performed concomitantly with CCE, and serum lactate level was reported. Acute respiratory distress syndrome (ARDS) was defined using the Berlin definition [12]. Plateau pressure, driving pressure and positive end-expiratory pressure were recorded, and the compliance of the respiratory system was calculated.

### CCE measurements

As previously described [Vieillard-Baron A, AJRCCM 2003], CCE was performed with continuous ECG and mechanical ventilation monitoring. An end-expiratory beat was defined as the last beat occurring before mechanical lung inflation. Among all variables prospectively recorded in the HemoPred cohort, we especially analyzed tricuspid annular plane systolic excursion (TAPSE), RV size, systolic pulmonary artery pressure (SPAP) and cardiac index (CI). TAPSE was measured at end-expiration with the M-mode study in an apical 4-chamber view as recommended [9]. RV size was also evaluated at end-expiration by the ratio of the RV/ LV end-diastolic area (EDA) in a transverse mid-esophageal view [13, 14]. Left ventricular stroke volume (LVSV) was calculated by combining the averaged aortic velocity time integral (AoVTI) by pulsed wave Doppler in the whole respiratory cycle with 2-D measurement of the related diameter [15], and CI was calculated as LVSV times heart rate indexed to the body surface area. SPAP was calculated at end-expiration based on the maximal velocity (Vmax) of the tricuspid regurgitation when available, as follows: SPAP = 4xVmax^2^ + CVP [16].

### Detection of fluid responsiveness

Passive leg raising (PLR), as technically validated [17], was performed to mimic fluid expansion. An increase of more than 10% in the AoVTI after 1-min PLR compared with baseline defined a significant increase in LV stroke volume [17, 18].

### Outcomes

The main outcome of the study was to evaluate the incidence of RV failure in septic shock patients.

The secondary outcomes included: 1) the relationship between RV failure and fluid responsiveness and 2) the performance of TAPSE in discriminating patients with and without RV failure.

### Statistical analysis

Continuous variables were reported as medians, and categorical data were expressed as numbers and percentages. Continuous variables were compared using a Mann–Whitney test or a Kruskal–Wallis test. Categorical data were compared using a Chi-square test or an exact Fisher test when necessary.

Three groups of patients were compared according to RV size and CVP, a good surrogate of venous congestion. Cutoff values of 0.6 for RV/LV EDA [14] and of 8 mmHg for CVP [19, 20] were considered. Patients in group 1 were defined as no RV dilatation (RV/LV ratio < 0.6). Patients in group 2 were defined as exhibiting RV dilatation (RV/LV ratio ≥ 0.6) and CVP < 8 mmHg; we then assumed that these patients did not have RV failure as no congestion was observed. Finally, patients in group 3 were defined as exhibiting RV dilatation (RV/LV ratio ≥ 0.6) associated with elevated CVP (≥ 8 mmHg) and were suspected to have RV failure. Spearman’s correlation coefficients were calculated in the three groups.

Correlation between TAPSE and RV/LV end-diastolic area was evaluated using Spearman’s correlation test. Rho and *p* values are provided. The paired Wilcoxon test was used to compare variations of CVP before and after PLR across the three groups. All the analyses were performed using R (R version 3.4.3 (2017-11-30)—"Kite-Eating Tree" Copyright (C) 2017 The R Foundation for Statistical Computing). A *p* value < 0.05 was considered significant.

## Results

**Fig. 1:**
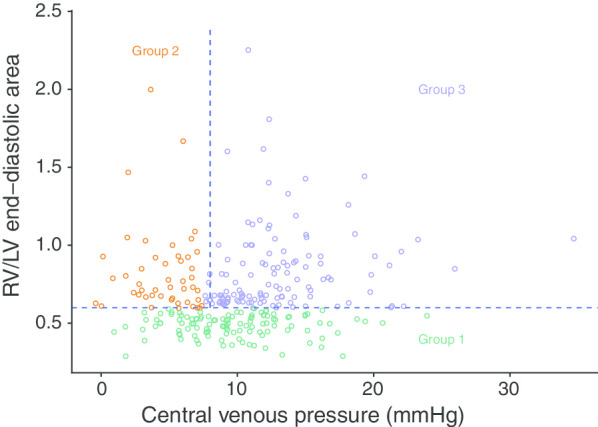
Individual values of CVP and RV/LV EDA among the 3 groups. Correlation (*ρ*) coefficients (*p* values in brackets) between CVP and RV/LV EDA were 0.023 (0.805), -0.102 (0.493) and 0.241 (0.008) in groups 1, 2 and 3, respectively

Among the 295 patients admitted for septic shock, 13 could not be classified according to RV/LV ratio (5 missing values) and CVP (8 missing values) (Additional file 1: Fig. S1). Thus, 282 patients were included in the analysis: 115 (41%) were in group 1 (no RV dilatation), 47 (17%) in group 2 (RV dilatation without failure) and 120 (42%) in group 3 (RV failure). Table 1 and Additional file 1: Table S1 report the main characteristics of the 3 groups. Briefly, body mass index and history of chronic respiratory failure were significantly increased in group 3, as was IAP. CI did not differ. No difference was observed for incidence of ARDS, and tidal volume was slightly higher in group 3. Patients in group 3 had more history of atrial fibrillation (18.3%) and 20.8% had atrial fibrillation at the time of the echocardiographic study, with no significant between-group difference, which did not allow the reporting of PPV. Figure 1 reports individual values for RV/LV EDA and CVP among the 3 groups. While no significant correlation was observed in groups 1 and 2, a significant relationship was observed in group 3 between both parameters (correlation ρ coefficient between CVP and RV/LV EDA was 0.023 (*p* = 0.805), − 0.102 (*p* = 0.493) and 0.241 (*p* = 0.008) in groups 1, 2 and 3, respectively).

**Table 1:** Baseline characteristics according to RV/LV EDA and CVP. Group 3 included patients suspected to have RV failure

	Group 1 (*N* = 115)	Group 2 (*N* = 47)	Group 3 (*N* = 120)	*p* value
Age, year	65.0 [56.0;74.5]	65.0 [55.5;74.5]	67.0 [59.0;77.0]	0.203
Weight	74.0 [66.5;83.0]	70.0 [59.5;80.0]	79.0 [69.5;90.0]	< 0.001
Height	172.0 [165.0;176.0]	170.0 [165.0;175.0]	170.0 [162.0;175.0]	0.294
Body mass index	24.9 [22.5;29.1]	24.5 [20.8;26.1]	27.8 [23.8;31.9]	< 0.001
SAPS2	55.0 [41.0;67.5]	57.0 [40.0;66.5]	59.0 [47.0;72.0]	0.113
SOFA score	10.0 [8.0;12.0]	9.0 [6.0;11.5]	10.0 [8.0;12.0]	0.527
*Hemodynamic parameters*				
Heart rate, bpm	108.5 [88.0;126.0]	105.0 [90.0;130.0]	103.0 [86.0;116.0]	0.056
Systolic arterial blood pressure, mmHg	120.0 [105.0;133.0]	96.0 [86.0;113.5]	108.5 [91.0;124.5]	< 0.001
Diastolic arterial blood pressure, mmHg	65.0 [55.5;74.0]	53.0 [47.0;63.5]	58.0 [51.0;68.0]	< 0.001
Mean arterial blood pressure, mmHg	82.0 [72.5;94.0]	70.0 [61.0;82.0]	74.0 [65.5;85.0]	< 0.001
Pulse pressure variation, %	9.0 [5.0;14.5]	13.0 [5.0;20.0]	8.0 [4.0;13.0]	0.127
Central venous pressure, mmHg	9.0 [7.0;12.0]	5.0 [3.0; 6.5]	12.0 [10.0;14.5]	0.000
Fluid expansion volume before echo, mL	2000.0 [1000.0;3696.5]	2000.0 [1427.5;4000.0]	2000.0 [1000.0;3500.0]	0.700
Arterial blood lactate level, mmol/L	2.3 [1.5; 4.0]	2.3 [1.4; 4.2]	2.2 [1.4; 3.8]	0.775
IAP, mmHg	10.0 [7.5;15.0]	8.5 [5.0;10.0]	11.0 [8.0;14.0]	0.002
Epinephrine	4 (4.0%)	2 (4.8%)	7 (7.1%)	0.619
Epinephrine dose, mg/h	0.9 [0.6; 1.1]	1.5 [1.0; 2.0]	2.0 [1.4; 2.6]	0.030
Norepinephrine	98 (98.0%)	40 (95.2%)	94 (94.9%)	0.487
Norepinephrine dose, mg/h	1.8 [1.0; 3.6]	1.9 [1.0; 2.8]	1.6 [0.8; 2.9]	0.655
Dobutamine	7 (7.0%)	6 (14.3%)	11 (11.1%)	0.368
Dobutamine dose, µg/kg/min	5.0 [5.0; 6.5]	5.0 [2.5; 5.0]	5.0 [2.5; 6.0]	0.637
*Echo parameters*				
Atrial fibrillation at the time of echo	17 (14.8)	5 (10.6)	25 (20.8)	0.247
*E*/*A* ratio	0.9 [0.7; 1.3]	0.9 [0.7; 1.2]	0.8 [0.7; 1.2]	0.630
Superior vena cava collapsibility, %	11.8 [5.3;25.0]	15.8 [6.3;28.6]	10.0 [5.0;25.8]	0.417
Inferior vena cava distensibility, %	5.3 [0.0;11.1]	5.9 [0.0;12.5]	4.5 [0.0; 9.5]	0.637
LVEF, %	51.0 [39.0;63.0]	53.0 [41.5;60.0]	51.0 [38.0;63.5]	0.995
RV/LV end-diastolic area	0.5 [0.4; 0.5]	0.7 [0.7; 0.9]	0.7 [0.7; 0.9]	0.000
Paradoxical septal motion	2 (1.7%)	4 (8.5%)	17 (14.2%)	0.001
Systolic pulmonary artery pressure, mmHg	41.0 [36.0;48.0]	38.5 [33.0;43.0]	45.0 [39.0;52.0]	0.016
CI, L/min/m^2^	3.8 [2.9;4.6]	3.8 [3.0;4.4]	3.4 [2.8;4.1]	0.392
TAPSE, mm	18.0 [15.0;22.0]	18.0 [15.0;21.0]	18.0 [14.0;21.0]	0.298
Fluid expansion after echo	50 (43.5%)	28 (59.6%)	37 (30.8%)	0.002
Aortic VTI, cm	19.4 [16.4;22.1]	16.8 [15.2;19.9]	20.0 [15.6;23.3]	0.185

Continuous variables are presented as the median [interquartile], while categorical variables are presented as *n* (%)

**Fig. 2:**
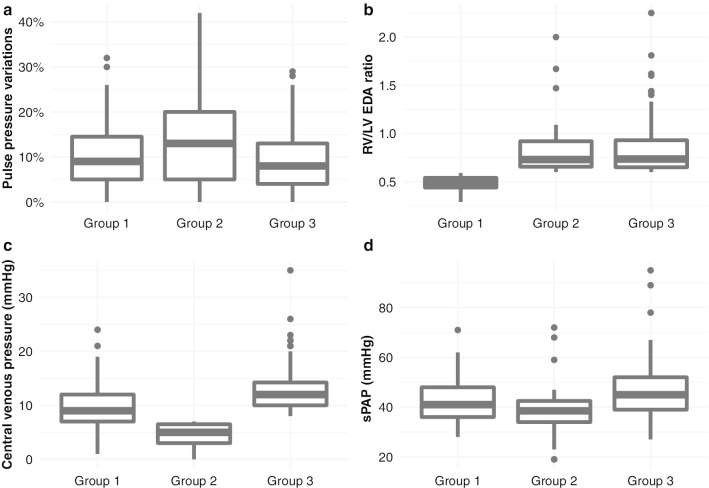
Hemodynamic parameters according to the three defined groups

No difference was observed for PPV between the 3 groups (Fig. 2a). As expected, RV/LV EDA was higher in groups 2 and 3 (0.7 [0.7;0.9]) compared to group 1 (0.5 [0.4;0.5], Fig. 2b). CVP was significantly different, 9 [7;12], 5 [3;6.5] and 12 mmHg [10;14.5] in groups 1, 2 and 3, respectively (Fig. 2c). Fourteen percent of patients in group 3 had paradoxical septal motion, compared to only 1.7% and 8.5% in groups 1 and 2, respectively (Table 1). Systolic pulmonary artery pressure was also higher in group 3 (45 mmHg [39;52], Fig. 2d).

**Fig. 3:**
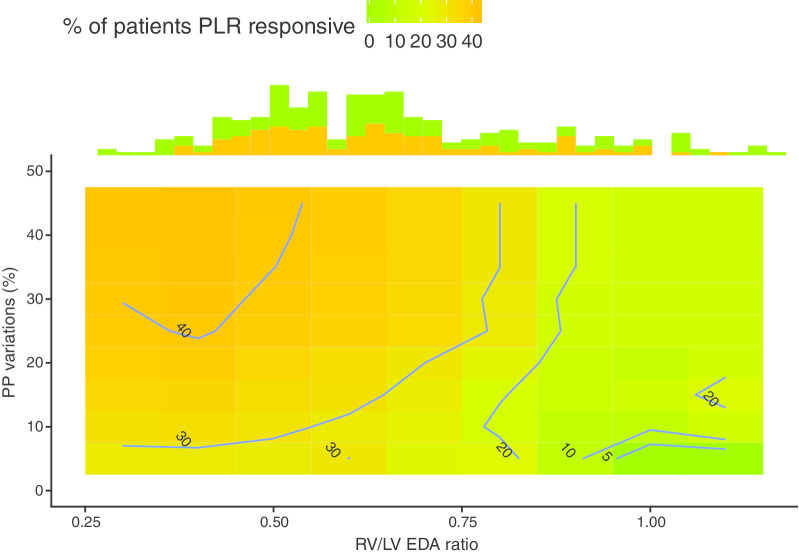
Proportion of patients responsive to passive leg raising according to pulse pressure variation and RV/LV end-diastolic area ratio. The colored scale represents the proportion of patients responsive to PLR in the analyzed cohort. The warmer the color, the higher the proportion of patients responsive to PLR. The blue lines show the thresholds that are indicative of the reader. The distribution of RV/LV end-diastolic area ratio is shown under the *x*-axis, according to response to PLR (PLR-responsive patients in orange and the non-responsive ones in green)

CVP significantly increased after PLR in the 3 groups by 1 [0,4], 3 [1,4.25] and 2 [0, 3] mmHg, respectively. As reported in Fig. 3, response to PLR according to PPV was substantially altered by RV size. Patients with significant PPV and no RV dilatation were more likely to respond to PLR than patients with a dilated right ventricle.

**Fig. 4:**
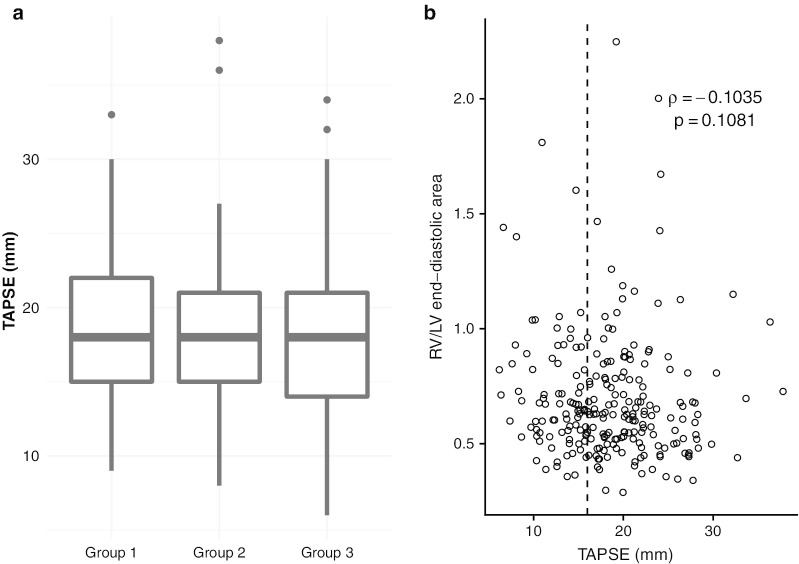
Distribution of TAPSE across the three defined groups (**a**) and its relationship with RV/LV end-diastolic area ratio (**b**)

Finally, Fig. 4a reports the distribution of TAPSE values (40 missing values, 14%) among the 3 groups. We did not observe any difference, with a median value in the normal range and a complete overlap between the 3 groups. Only 38/104 (36.5%) patients in group 3 had a TAPSE < 16 mm and no relationship (rho = -0.104, *p* = 0.108) was observed between TAPSE and RV size (Fig. 4b).

## Discussion

### Main results

RV failure was frequent (42% of cases) when defined by the association of RV dilatation (RV/LV EDA ≥ 0.6) with systemic congestion (CVP ≥ 8 mmHg). RV size modified the response to a reversible fluid challenge (PLR), as higher RV dilatation was associated with less likelihood of fluid responsiveness for a given PPV. TAPSE was also not accurate enough to detect RV failure since overlap was observed between the 3 groups and around 60% of patients with RV failure had normal values.

### Definition of RV failure

The current definition is only physiologically based and has no clear cutoff values for RV size and CVP [3–5], CVP being used here as a surrogate of systemic congestion. Indeed, RV failure is now recognized as a state where the right ventricle is substantially dilated and associated with systemic congestion. In our study, 43.3% (52/120) of patients considered to show RV failure had an RV/LV EDA ratio greater than or equal to 0.8, which was recently shown to be a strong indicator of RV failure [21]. Applying a cutoff of 8 mmHg for CVP is supported by a clinical study reporting a worse outcome when CVP is above this value [6]. Besides, the Surviving Sepsis Campaign does not recommend more fluid expansion in patients with persistent shock when CVP is higher than 8 mmHg [20]. Finally, Mullens et al. reported that among patients with advanced decompensated heart failure, those with higher CVP at admission or who did not achieve a CVP < 8 mmHg after treatment were more likely to develop worsening of renal failure by venous congestion [19]. Interestingly, we did not find a correlation between CVP and RV size in patients without RV failure (groups 1 and 2), which is to be expected as CVP is known not to reflect RV filling in normal conditions and a normal right ventricle works under its stressed volume [22, 23]. However, we found a correlation in patients with RV failure, which probably reflects that the right ventricle is stressed.

### RV and sepsis

Septic cardiomyopathy affects the right ventricle [24]. In a small cohort of 40 mechanically ventilated septic shock patients, we reported RV dilatation in 32.5% of cases [25]. We did not report CVP in these specific patients, but the mean CVP in the whole population was elevated, i.e., around 13 mmHg, and patients had significant PPV. ARDS has been clearly reported as a risk factor for RV failure [13, 26], while in the present cohort we did not find a higher incidence of ARDS in these patients. However, we found a certain degree of pulmonary hypertension that could be related to an increase in BMI with a history of chronic respiratory failure. Patients with RV failure were also ventilated with a higher tidal volume. This association of RV function impairment related to sepsis with positive pressure ventilation and pulmonary hypertension may explain RV failure and the higher incidence of paradoxical septal motion we observed in these patients.

### Clinical application

Our results draw the physician’s attention to the fact that PPV is not systematically associated with fluid responsiveness but also with RV failure that is an obvious situation with no benefit of fluid expansion. A significant PPV was less likely to predict a positive response to PLR in the case of RV dilatation, and this effect was more pronounced when the dilatation increased. Based on our results, we can only report that patients with RV failure, as defined in our study, will respond to fluids in only 30% of cases and so that the risk of giving useless and even harmful fluids is high. To decide to give fluids, physicians have then to suspect a potential high clinical benefit of a positive response. In patients with RV/LV > 0.6 but CVP < 8, we could more clearly suggest that fluids may unmask RV failure, as said by the reviewer, and then that fluid expansion, if considered, has to be done with caution (low volume to test the system) and by monitoring CVP and RV size (Mercat A CCM 1999, Konstantinides SV Eur Heart J 2014). This would highlight the need of a repeated hemodynamic evaluation using echocardiography to evaluate RV size when PPV are significant, combined with CVP monitoring which may diagnose congestion, part of RV failure. We demonstrated that RV failure was frequently observed in septic shock patients and echo is the best and easiest way to figure it out. However, our observations with their clinical applications require further validation before to be recommended in daily systematic routine.

### Limitations

Our study has some limitations. First, this is a post hoc analysis of the HemoPred study, which was not specifically designed for such a goal. However, HemoPred was a multicenter study with a very low number of missing values. Second, Beurton et al. suggested than PLR could be of limited accuracy in predicting fluid responsiveness when IAP is above 12 mmHg [27]. If so, it could clearly alter our result regarding the impact of RV size on fluid responsiveness according to PPV. However, a systematic review and meta-analysis including 21 studies and 991 unselected patients done by the same team reported this technique as near perfect [18]. Median values of IAP in our study were lower than 12 mmHg in our 3 groups. However, as we did not directly fill our patients because we considered it would be unethical, we cannot completely exclude such a limitation, even though CVP increased significantly after PLR, suggesting that the maneuver efficiently induced a reversible fluid challenge [28]. Third, it would have been interesting to evaluate other echo indices of RV function to confirm our results. Despite unavailable, we believe this would not change regarding the high correlation between TAPSE and the other RV systolic function indices as the S’ wave.

## Conclusions

We report in a large population of ventilated septic shock patients that when RV failure is defined using critical care echocardiography (RV/LV EDA ≥ 0.6) and a surrogate of venous congestion (CVP ≥ 8 mmHg), it was frequently observed (42%) with a negative effect on the response to a fluid challenge even in the case of significant PPV. TAPSE was unable to differentiate between patients with or without RV failure. Future studies should validate our results and our definition of RV failure in a larger population in order to improve hemodynamic management in these critically ill patients.

## Supplementary information

**Additional file 1**. Electronic supplementary material.

## Data Availability

Data are not available.
