# Supplementary material for: Right ventricular failure in septic shock: characterization, incidence and impact on fluid responsiveness
Source: Crit Care. 2020 Nov 1;24:630. doi: 10.1186/s13054-020-03345-z (PMC7603714; doi:10.1186/s13054-020-03345-z)

Right ventricular failure in septic shock
Characterization, incidence and impact on fluid-responsiveness.

Antoine Vieillard-Baron[1, 2, 3], MD, PhD, Amélie Prigent [1, 2], MD, Xavier Repesse [1], MD, Marine Goudelin, MD, Gwenaël Prat [4], MD, Bruno Evrard[5], MD, Cyril Charron [1], MD, Philippe Vignon [5, 6,7], MD, PhD, Guillaume Geri [1, 2, 3], MD, PhD

Additional file

Table S1: Baseline characteristics according to RV/LV EDA and CVP. Group 3 included patients suspected to have RV failure.

Continuous variables are presented as the median [interquartile], while categorical variables are presented as n (%).

	**Group 1 (N=115)**	**Group 2 (N=47)**	**Group 3 (N=120)**	**P value**
**Medical history**				
Chronic heart failure	19 (16.5%)	6 (12.8%)	21 (17.5%)	0.756
Chronic resp. failure	11 (9.6%)	8 (17.0%)	25 (20.8%)	0.056
Atrial fibrillation	9 (7.8%)	5 (10.6%)	22 (18.3%)	0.049
Recent abdominal surgery	40 (34.8%)	15 (31.9%)	32 (26.7%)	0.398
**Respiratory parameters**				
ARDS	35 (30.4%)	16 (34.0%)	40 (33.3%)	0.858
Positive end-expiratory pressure, cmH_2O	6 [5; 8]	5 [0; 6]	5 [4; 8]	<0.001
Tidal volume, mL	7.2 [6.6; 8.0]	7.7 [7.0; 8.4]	7.8 [7.1; 8.8]	0.001
Driving pressure, cmH_2O	13.0 [10.0;16.0]	14.0 [11.0;15.0]	14.0 [12.0;17.0]	0.137
**Laboratory parameters**				
Arterial pH	7.3 [7.2; 7.4]	7.3 [7.2; 7.4]	7.3 [7.2; 7.4]	0.894
PaO_2, mmHg	105.0 [80.0;134.0]	92.0 [73.5;124.0]	90.5 [70.0;138.0]	0.162
$PaCO_2$, mmHg	40.0 [34.0;46.0]	40.0 [35.5;48.0]	42.0 [35.0;50.5]	0.754
SaO_2, %	98.0 [95.0;99.0]	97.0 [92.0;98.0]	97.0 [93.0;99.0]	0.135
Base excess, mmol/L	-5.2 [-9.7;-2.1]	-6.3 [-10.7;-1.1]	-5.2 [-10.3;-1.0]	0.887
Bicarbonates, mmol/L	20.1 [16.9;23.4]	20.0 [15.9;23.5]	20.8 [16.0;25.0]	0.659

Figure S1. Flowchart of the study

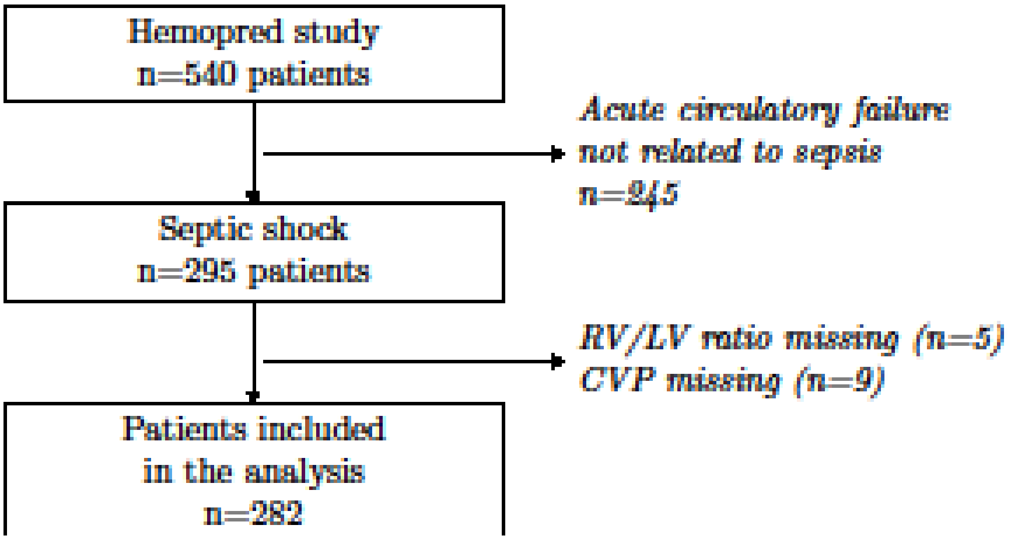

Supplement: Supplementary file 1 — Additional file 1. Electronic supplementary material. [file 13054_2020_3345_MOESM1_ESM.pdf]
